# Supplementary material for: Salmonella exploits membrane reservoirs for invasion of host cells
Source: Nat Commun. 2024 Apr 10;15:3120. doi: 10.1038/s41467-024-47183-x (PMC11006906; doi:10.1038/s41467-024-47183-x)

Supplementary Information

Salmonella exploits membrane reservoirs for invasion of host cells

Hongxian Zhu[1,2], Andrew M. Sydor[1], Kirsten C. Boddy[1,3], Etienne Coyaud[4,5], Estelle M.N. Laurent[4,5], Aaron Au[6], Joel M.J. Tan[1], Bing-Ru Yan[1], Jason Moffat[2,6,7], Aleixo M. Muise[1,8,9,10], Christopher M. Yip[6,8], Sergio Grinstein[1,3,8], Brian Raught[4,11], John H. Brumell[1,2,3,10,*]

[1]Cell Biology Program, Hospital for Sick Children, Toronto, ON, M5G 1X8, Canada
[2]Department of Molecular Genetics, University of Toronto, Toronto, ON M5S 1A8, Canada
[3]Institute of Medical Science, University of Toronto, Toronto, ON, M5S 1A8, Canada
[4]Princess Margaret Cancer Centre, University Health Network, Toronto, ON, M5S 1A8, Canada.
[5]Univ. Lille, Inserm, CHU Lille, U1192 - Protéomique Réponse Inflammatoire Spectrométrie de Masse - PRISM, F-59000 Lille, France.
[6]Institute of Biomedical Engineering, University of Toronto, Toronto, ON, M5S3G9, Canada
[7]Genetics and Genome Biology Program, Hospital for Sick Children, Toronto, ON, M5G 1X8, Canada
[8]Department of Biochemistry, University of Toronto, Toronto, ON M5S1A8, Canada
[9]Division of Gastroenterology, Hepatology and Nutrition, Department of Pediatrics, Hospital for Sick Children, Toronto, ON, M5G1X8, Canada
[10]SickKids IBD Centre, Hospital for Sick Children, Toronto, ON, M5G 0A4, Canada
[11]Department of Medical Biophysics, University of Toronto, Toronto, ON M5S 1A8, Canada

[*]Corresponding author:
John H. Brumell, Cell Biology Program, Hospital for Sick Children, 686 Bay Street PGCRL, Toronto, ON, M5G 0A4, Canada Tel: 416-813-7654 ext. 303555. E-mail: john.brumell@sickkids.ca

Key Words: Bacterial pathogenesis, *Salmonella*, membrane reservoir, host-pathogen interaction, RAB10, GTPase, exocyst

Supplementary Figures

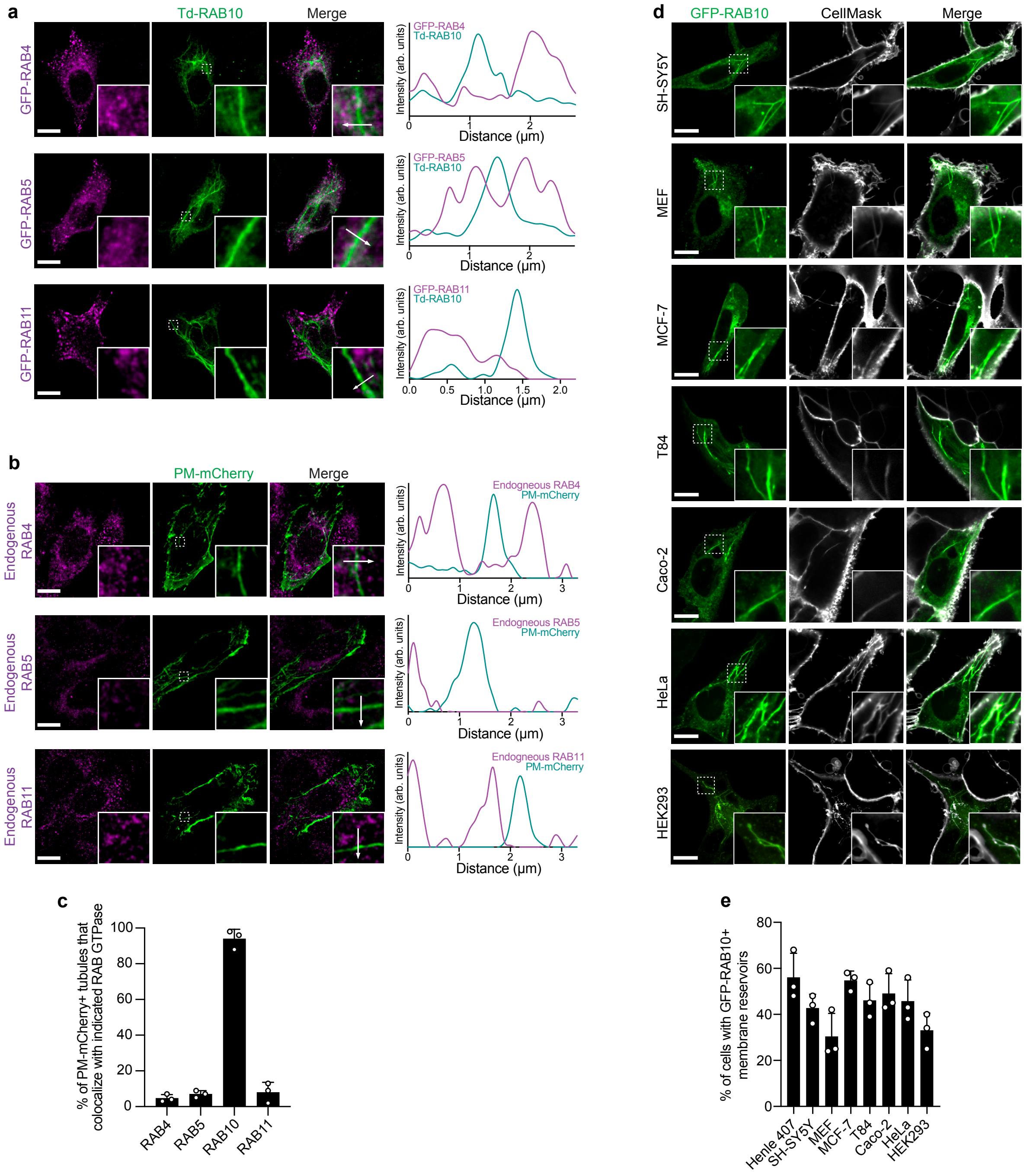

Supplementary Figure 1: RAB10 localizes to membrane reservoirs in multiple cell types. **a,** Representative images and line plot profiles (white arrows in insets) of WT Henle 407 cells transfected with Td-RAB10 and indicated GFP-tagged RAB GTPase. MCC values for GFP-RAB4 and Td-RAB10 are M1=0.06±0.03 and M2=0.06±0.02. For GFP-RAB5 and Td-RAB10, M1=0.08±0.02 and M2=0.08±0.02. For GFP-RAB11 and Td-RAB10, M1=0.05±0.03 and M2=0.05±0.02. **b**, Representative images and line plot profiles (white arrows in insets) of WT Henle 407 cells transfected with PM-mCherry and stained with antibody against indicated RAB

GTPase. MCC values for endogenous RAB4 and PM-mCherry are M1=0.06±0.03 and M2=0.05±0.02. For endogenous RAB5 and PM-mCherry, M1=0.05±0.03 and M2=0.07±0.04. For endogenous RAB11 and PM-mCherry, M1=0.04±0.03 and M2=0.06±0.02. **c**, Quantifications of the percentage of PM-mCherry+ tubules that colocalize with indicated RAB GTPase. n=3 independent experiments with 100 PM-mCherry+ tubules examined in each experiment. **d** and **e**, Representative images (**d**) and quantifications (**e**) of $RAB10^{+}$ tubules in indicated cell lines that are $CellMask^{+}$ and identified as membrane reservoirs. n=3 independent experiments with 100 cells examined in each experiment. Source data are provided as a Source Data file.

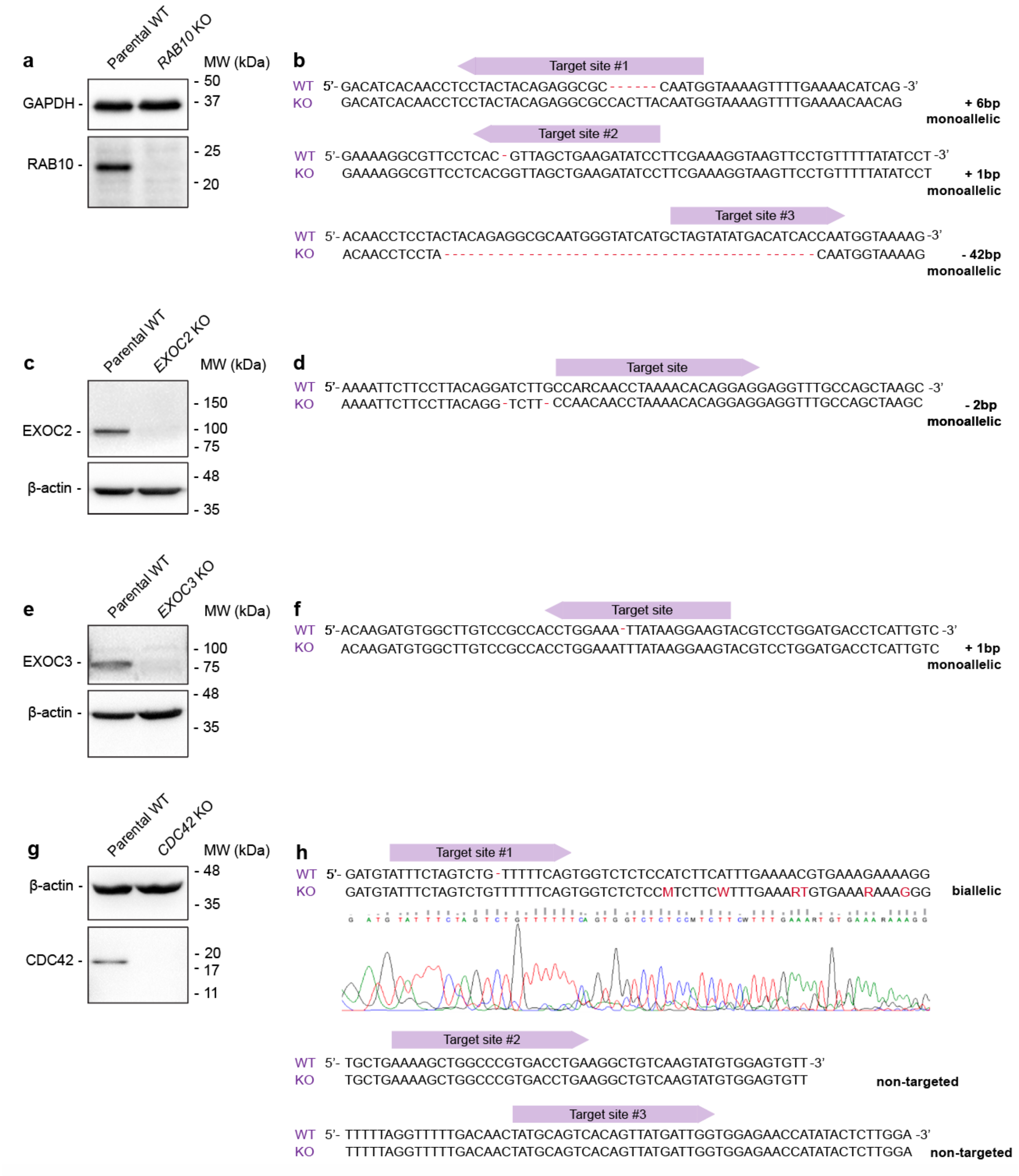

Supplementary Figure 2: Verifications of CRISPR/Cas9-mediated knockout cell lines.
a, Western blot confirming the CRISPR–Cas9-mediated deletion of RAB10. Total lysates from control sgRNA- or RAB10-specific sgRNA-treated Henle 407 cells were immunoblotted against RAB10. GAPDH served as a loading control. **b**, Sequencing results around RAB10-specific sgRNA-targeted site. The details of introduced mutations were also described. **c**, Western blot confirming the CRISPR–Cas9-mediated deletion of EXOC2. Total lysates from control sgRNA- or EXOC2-specific sgRNA-treated Henle 407 cells were immunoblotted against EXOC2. β-actin

served as a loading control. **d**, Sequencing results around EXOC2-specific sgRNA-targeted site. The details of introduced mutation was also described. **e**, Western blot confirming the CRISPR–Cas9-mediated deletion of EXOC3. Total lysates from control sgRNA- or EXOC3-specific sgRNA-treated Henle 407 cells were immunoblotted against EXOC3. β-actin served as a loading control. **f**, Sequencing results around EXOC3-specific sgRNA-targeted site. The details of introduced mutation was also described. **g**, Western blot confirming the CRISPR–Cas9-mediated deletion of CDC42. Total lysates from control sgRNA- or CDC42-specific sgRNA-treated Henle 407 cells were immunoblotted against CDC42. β-actin served as a loading control. **h**, Sequencing results and chromatogram (sgRNA #1) around CDC42-specific sgRNA-targeted site. The details of introduced mutation was also described. Source data are provided as a Source Data file.

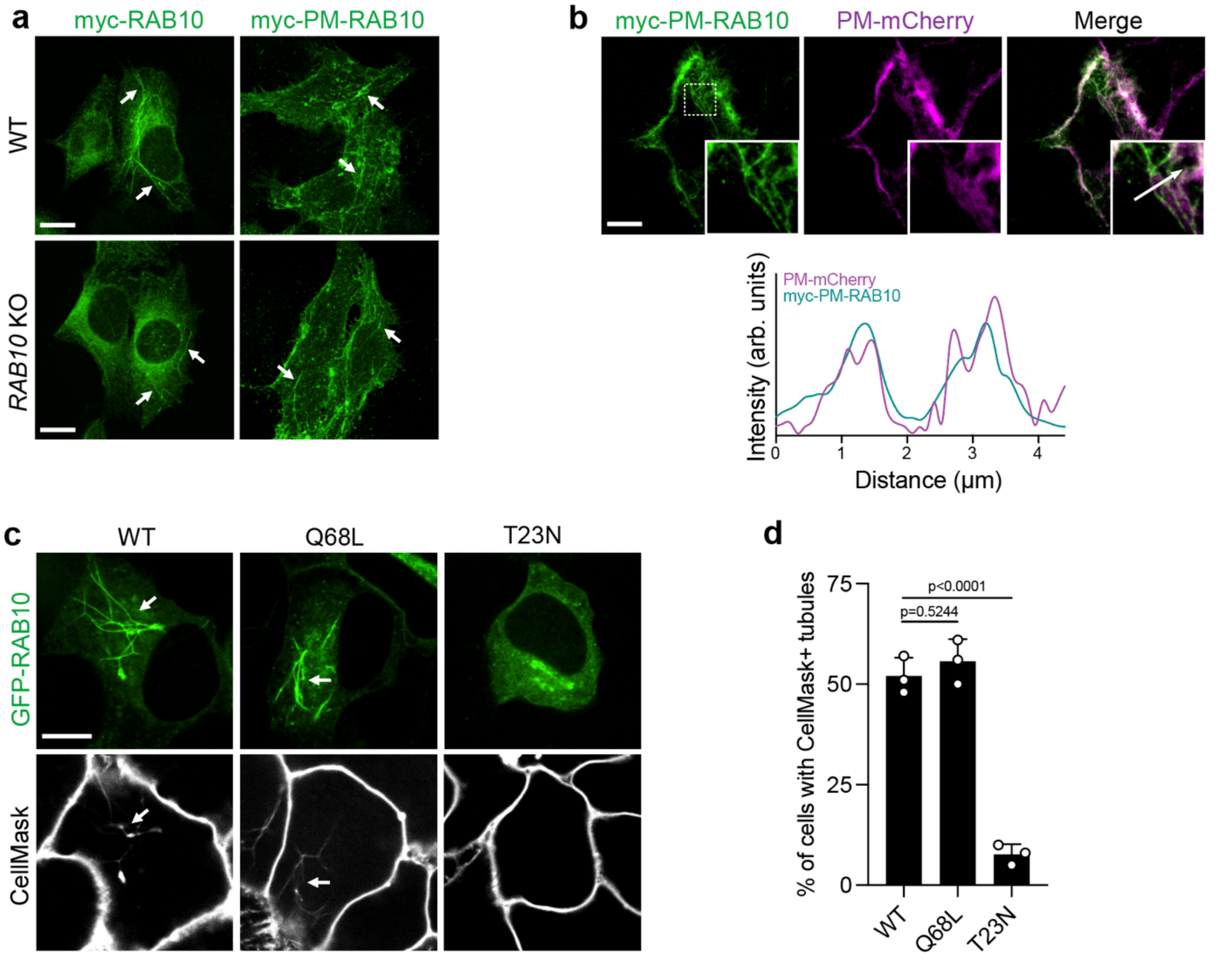

Supplementary Figure 3: RAB10's local activity is required to generate membrane reservoirs.

a, Representative images of WT or *RAB10* KO Henle 407 cells transfected with myc-RAB10 or myc-PM-RAB10. Cells were stained with an antibody against the myc-tag. Arrows indicate $RAB10^{+}$ membrane reservoirs. **b**, Representative images of *RAB10* KO Henle 407 cells transfected with myc-PM-RAB10 and PM marker (PM-mCherry), and stained with antibody against the myc-tag. Insets and line plot profiles of the white arrow in the inset depict myc-PM-RAB10's colocalization with PM-mCherry. MCC values for myc-PM-RAB10 and PM-mCherry are M1=0.60±0.06 and M2=0.48±0.11. **c** and **d**, Representative images (**c**) and quantifications (**d**) of $CellMask^{+}$ tubules in WT Henle 407 cells transfected with GFP-RAB10 WT, Q68L or T23N mutants. White arrows indicate $RAB10^{+}$ membrane reservoirs that are positive for either CellMask or a PM marker. n=3 independent experiments with 100 cells examined in each experiment. Data shown are means ± standard error of the mean (S.D.). *P* value was calculated using (**d**) one-way ANOVA. Scale bars, 10 µm. Source data are provided as a Source Data file.

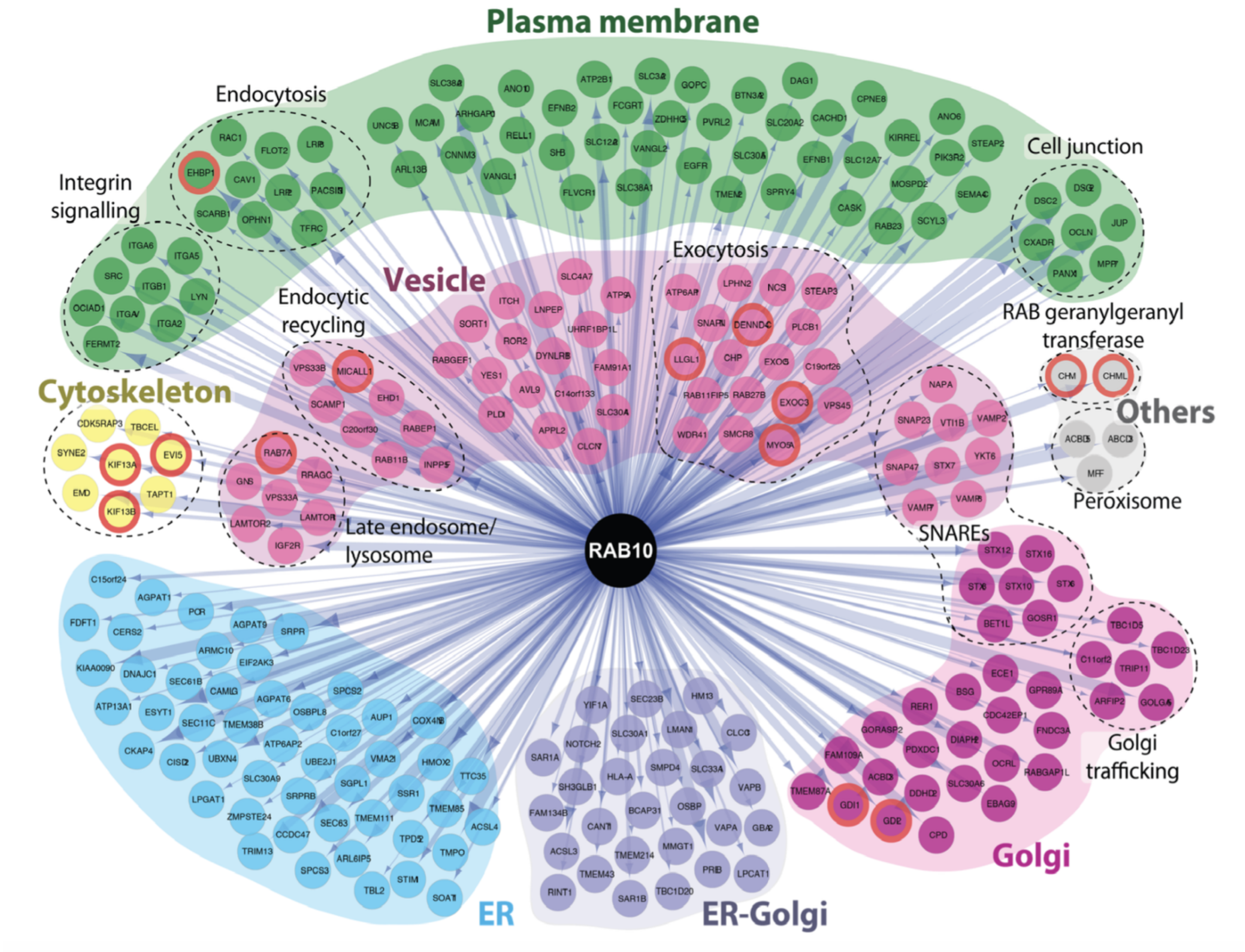

Supplementary Figure 4: RAB10 interactors are identified from BioID screen and categorized based on known localizations.

The proximity-based interactome report from RAB10 WT BioID. Profile of BioID hits from WT FLAG-RAB10 stable Flp-In T-REx 293 cells. Each hit from the BioID screen is organized by known localization and function. The thickness of each line is proportional to peptide counts and a red circle denotes known interactors. Red circles indicate RAB10's known effectors. Data represents four technical replicates from two biological repeats of the experiment.

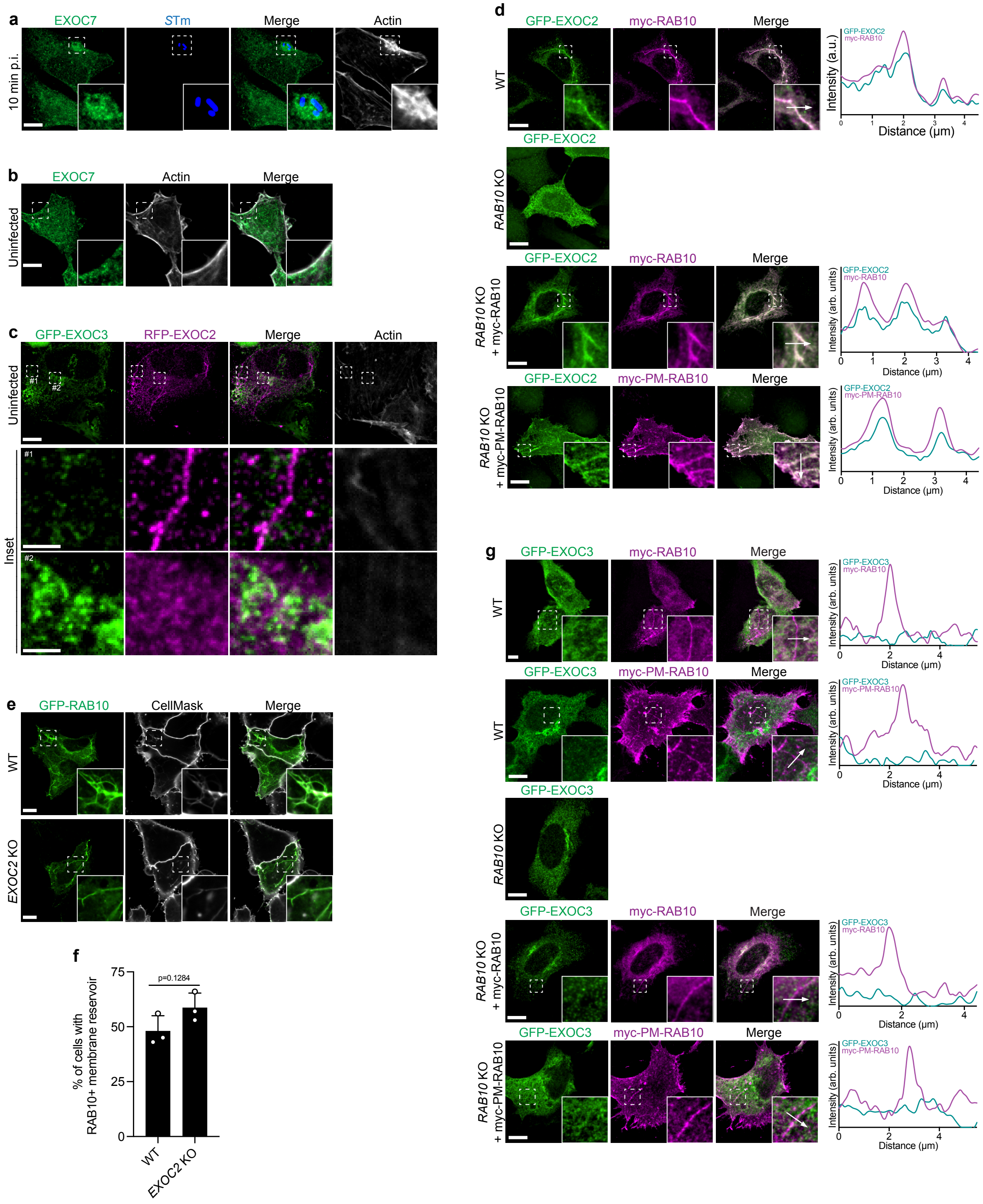

Supplementary Figure 5: Exocyst component EXOC2 but not EXOC3 localizes to RAB10$^+$ membrane reservoirs prior to infection.

a, Representative images depicting EXOC7's recruitment to *S*Tm invasion sites. WT Henle 407 cells were infected with WT *S*Tm. Cells were fixed and imaged at 10 min post-infection. Cells were stained with antibody against EXOC7. **b**, Representative images depicting EXOC7's

localization in normal growth condition. WT Henle 407 cells were were fixed and stained with antibody against EXOC7 and Phalloidin actin staining dye. Data are representative of three independent experiments. c, Representative images of WT Henle 407 cells transfected with GFP-EXOC3 and RFP-EXOC2 constructs. Inset 'i' indicates a ROI with EXOC2 localization to tubular structures while inset 'ii' indicates a ROI of a EXOC3 localization to peri-nuclear region. **d**, WT, *RAB10* KO or *RAB10* KO Henle 407 cells with myc-RAB10 or myc-PM-RAB10 overexpression were transfected with GFP-EXOC2 and fixed and stained with antibody against myc-tag. Representative images depicting EXOC2's localization on RAB10$^+$ membrane reservoirs with the complemented RAB10 (either total or PM-targeted RAB10) expression in *RAB10* KO cells. MCC values for GFP-EXOC2 and myc- RAB10 in WT cells are M1=0.39±0.12and M2=0.47±0.05. For GFP-EXOC2 and myc-RAB10 in *RAB10* KO cells complemented with myc-RAB10, M1=0.45±0.08 and M2=0.44±0.15. For GFP-EXOC2 and myc-PM-RAB10 in *RAB10* KO cells complemented with myc-PM-RAB10, M1=0.57±0.08 and M2=0.54±0.10. **e** and **f**, Representative images (**e**) and quantifications (**f**) of percentage of cell with RAB10$^+$ membrane reservoirs. WT and *EXOC2* KO Henle 407 cells were transfected with GFP-RAB10, and then fixed and stained with CellMask. n=3 independent experiments with 100 cells examined in each experiment. **g**, WT, *RAB10* KO Henle 407 cells or *RAB10* KO Henle 407 cells with myc-RAB10 or myc-PM-RAB10 overexpression were transfected with GFP-EXOC3 and fixed and stained with antibody against myc-tag. Representative images depicting EXOC3's absence from RAB10$^+$ (either total or PM-targeted RAB10) membrane reservoirs in all conditions. MCC values for GFP-EXOC3 and myc-RAB10 in WT cells are M1=0.05±0.02 and M2=0.04±0.02. For GFP-EXOC3 and myc-PM-RAB10 in WT cells, M1=0.07±0.03 and M2=0.06±0.02. For GFP-EXOC3 and myc-RAB10 in *RAB10* KO cells complemented with myc-RAB10, M1=0.05±0.02 and M2=0.06±0.02. For GFP-EXOC3 and myc-PM-RAB10 in *RAB10* KO cells complemented with myc-PM-RAB10, M1=0.07±0.03 and M2=0.06±0.03. Data shown are means ± S.D.. *P* value was calculated using (**f**) two tailed unpaired *t*-test. Scale bars, 10 μm. Source data are provided as a Source Data file.

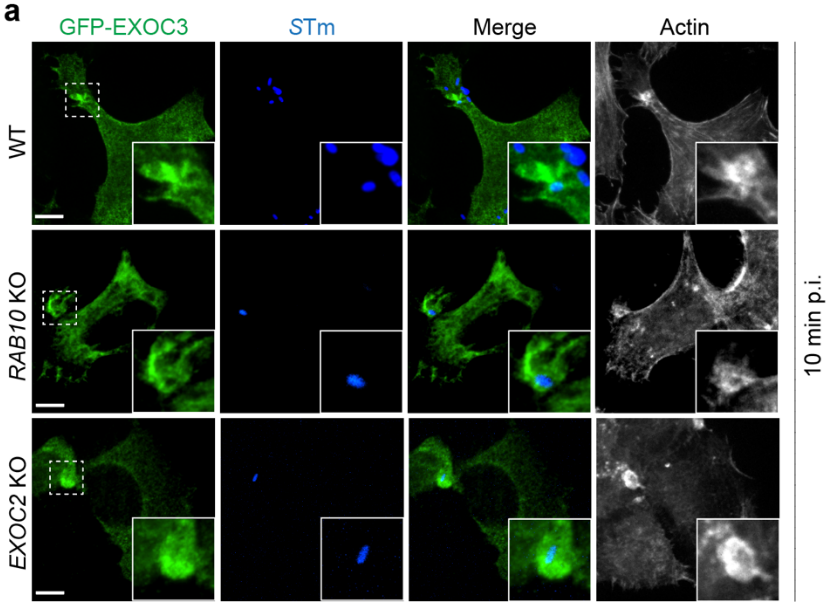

Supplementary Figure 6: EXOC3 recruitment to *S*Tm invasion sites is independent of RAB10 or EXOC2 expression.
a, Representative images of EXOC3 recruitment to *S*Tm invasion sites. WT, *RAB10* KO or *EXOC2* KO Henle 407 cells were transfected with GFP-EXOC3, and then infected with WT *S*Tm. Cells were fixed and imaged at 10 min post-infection. Data are representative of three independent experiments.

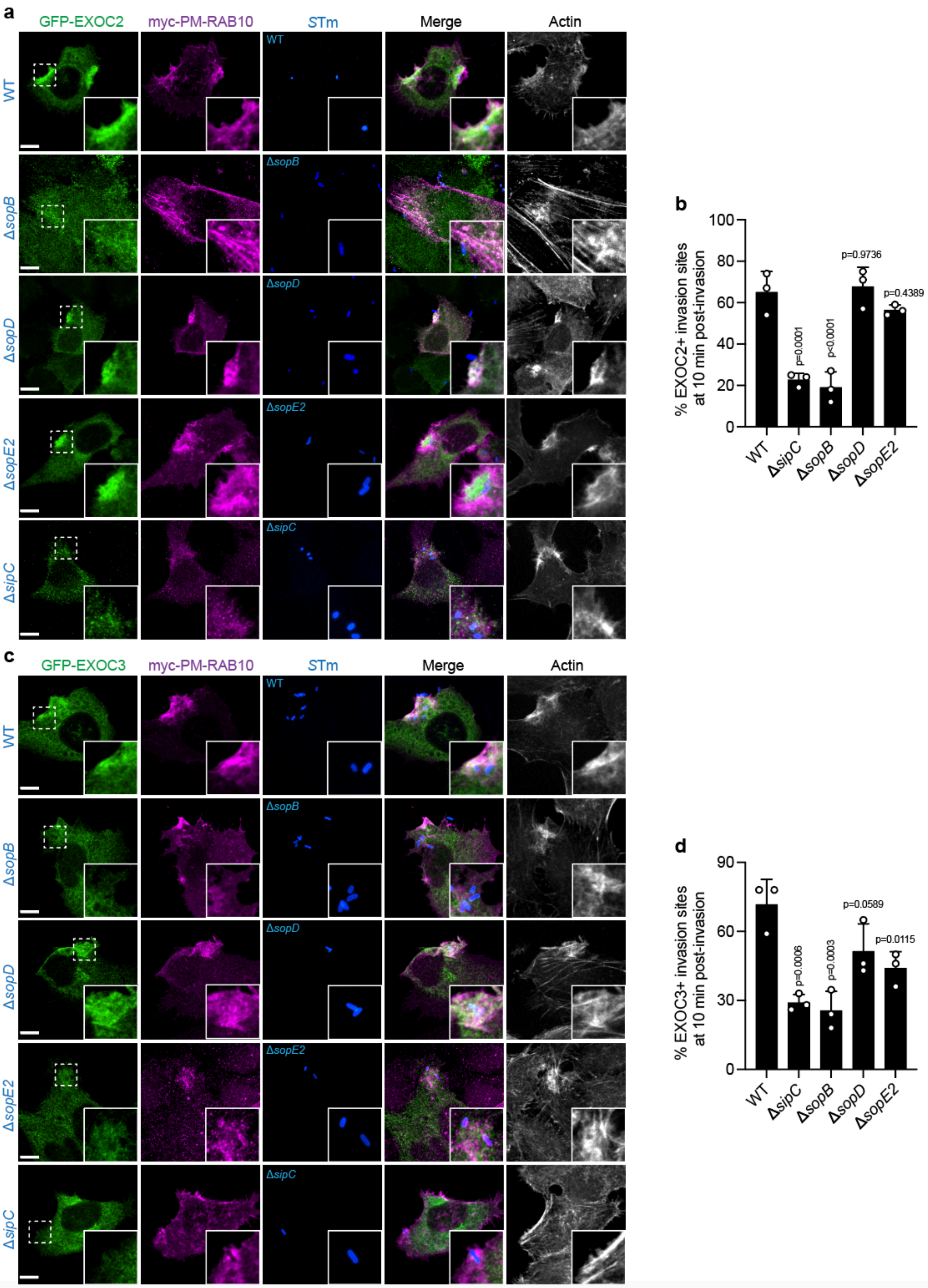

Supplementary Figure 7: SPI-1 T3SS effectors act cooperatively to recruit the exocyst complex to *S*Tm invasion sites.

a and **b**, Representative images (**a**) and quantifications (**b**) of EXOC2 recruitment to *S*Tm invasion sites. WT Henle 407 cells were transfected with GFP-EXOC2 and myc-PM-RBA10, and then infected with WT *S*Tm or mutated *S*Tm strain carrying indicated effector deletion. Cells were fixed and imaged at 10 min post-infection. n=3 independent experiments with 100 invasion sites examined in each experiment. **c** and **d**, Representative images (**c**) and quantifications (**d**) of EXOC3 recruitment to *S*Tm invasion sites. WT Henle 407 cells were transfected with GFP-EXOC3 and myc-PM-RBA10, and then infected with WT *S*Tm or mutated *S*Tm strain carrying indicated effector deletion. Cells were fixed and imaged at 10 min post-infection. n=3 independent experiments with 100 invasion sites examined in each experiment. Data shown are means ± S.D.. *P* value was calculated using (**b** and **d**) one-way ANOVA. Scale bars, 10 µm. Source data are provided as a Source Data file.

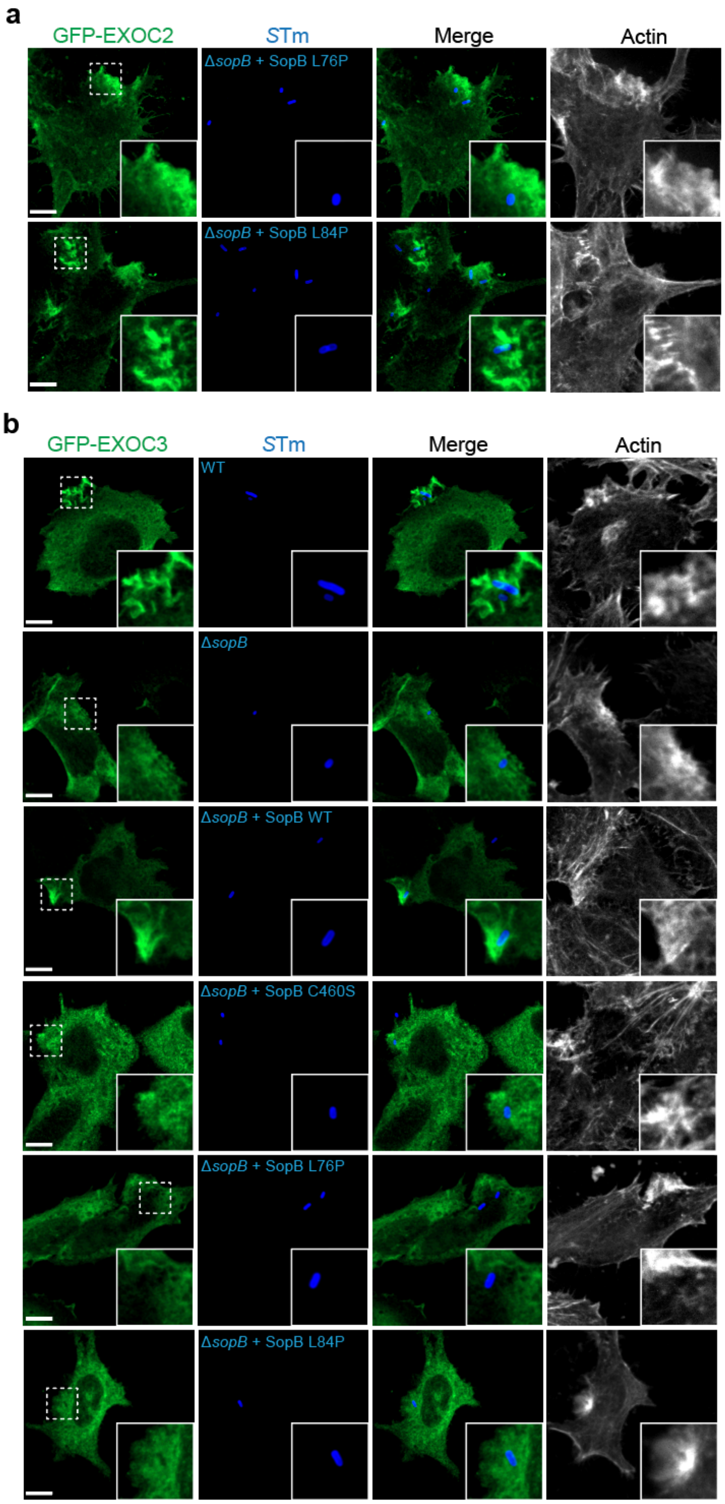

Supplementary Figure 8: SopB recruits EXOC2 and EXOC3 to *S*Tm invasion sites via independent pathways.

a, Representative images of EXOC2 recruitment to *S*Tm invasion sites. WT Henle 407 cells were transfected with GFP-EXOC2, and then infected with mutated *S*Tm strain carrying indicated SopB mutation. Cells were fixed and imaged at 10 min post-infection and actin and *S*Tm staining

were used to identify invasion sites. **b**, Representative images of EXOC3 recruitment to *S*Tm invasion sites. WT Henle 407 cells were transfected with GFP-EXOC3, and then infected with WT *S*Tm or mutated *S*Tm strain carrying indicated SopB mutation. Cells were fixed and imaged at 10 min post-infection. Data are representative of three independent experiments.

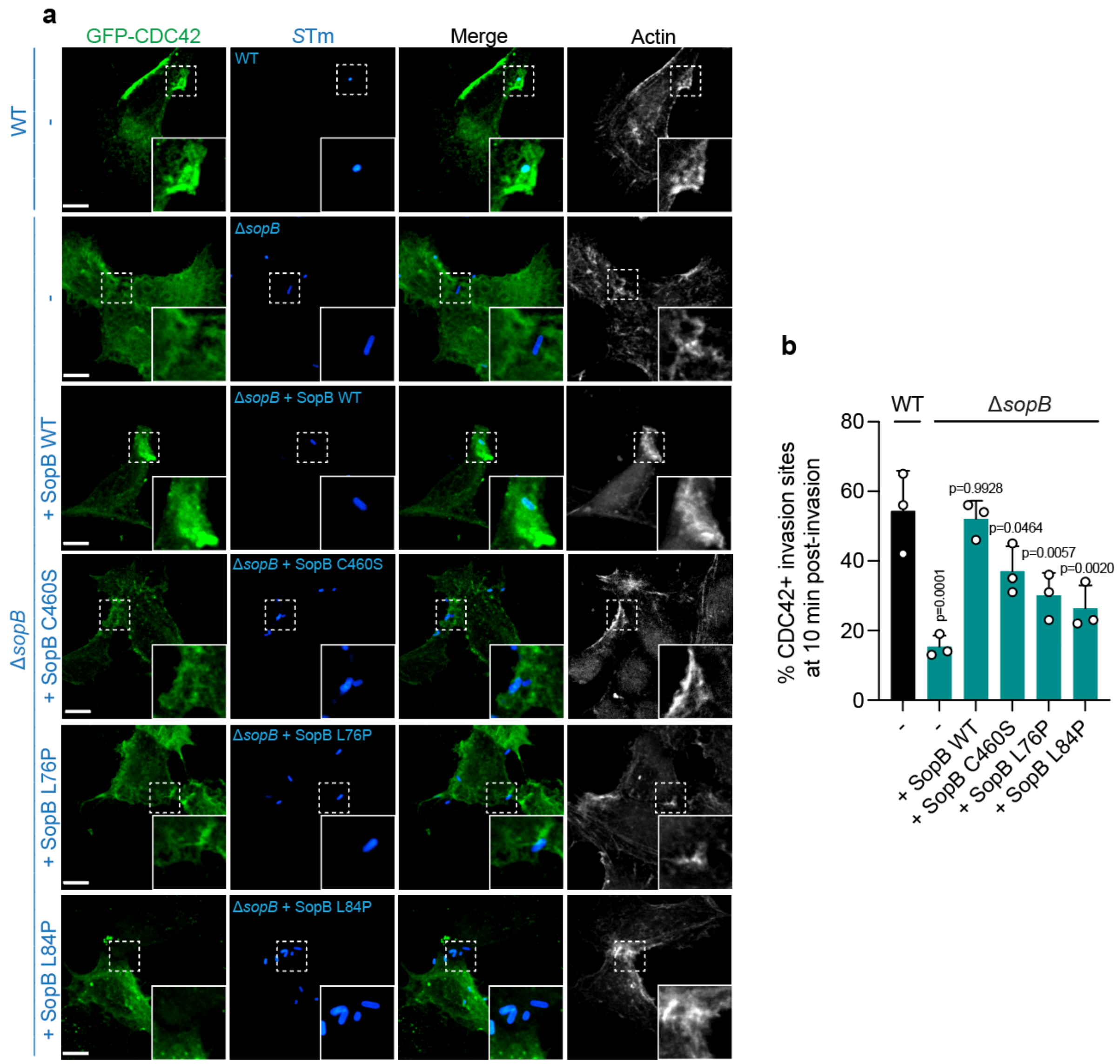

Supplementary Figure 9: Both catalytic activity and direct binding are required for SopB-mediated CDC42 recruitment to *S*Tm invasion sites.

a and **b**, Representative images (**a**) and quantifications (**b**) of CDC42 recruitment to *S*Tm invasion sites. WT Henle 407 cells were transfected with GFP-CDC42, and then infected with mutated *S*Tm strain carrying indicated SopB mutation. Cells were fixed and imaged at 10 min post-infection. n=3 independent experiments with 100 invasion sites examined in each experiment. Data shown are means ± S.D.. *P* value was calculated using one-way ANOVA. Scale bars, 10 μm. Source data are provided as a Source Data file.

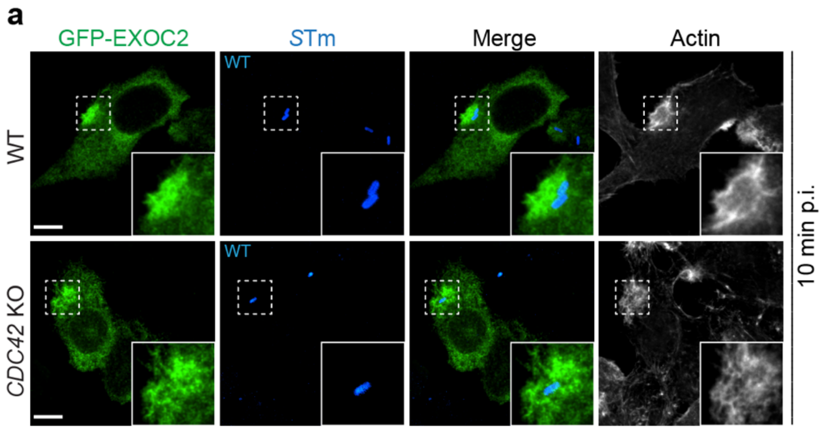

Supplementary Figure 10: EXOC2 recruitment to *S*Tm invasion sites is independent of CDC42 expression.

a, Representative images of EXOC2 recruitment to *S*Tm invasion sites. WT or *CDC42* KO Henle 407 cells were transfected with GFP-EXOC2, and then infected with WT *S*Tm. Cells were fixed and imaged at 10 min post-infection. Data are representative of three independent experiments.

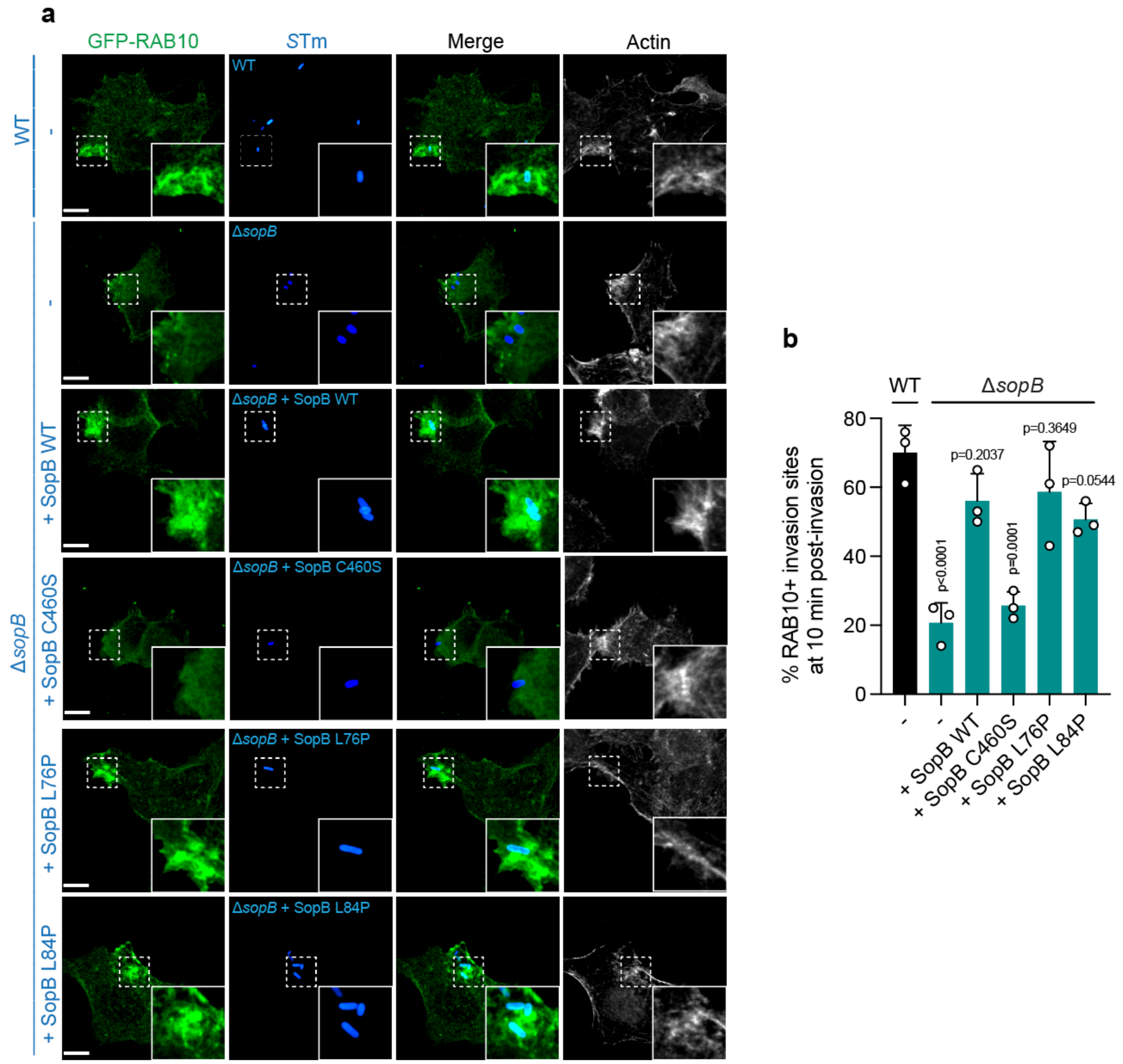

Supplementary Figure 11: SopB recruits RAB10 to invasion sites independent of SopB-CDC42 binding.

a and **b**, Representative images (**a**) and quantifications (**b**) of RAB10 recruitment to *S*Tm invasion sites. WT Henle 407 cells were transfected with GFP-RAB10, and then infected with mutated *S*Tm strain carrying indicated SopB mutation. Cells were fixed and imaged at 10 min post-infection. n=3 independent experiments with 100 invasion sites examined in each experiment. Data shown are means ± S.D.. *P* value was calculated using one-way ANOVA. Scale bars, 10 µm. Source data are provided as a Source Data file.

Supplementary Tables

Supplementary Table 1: STR profiling of Henle 407 cells.

The Centre for Applied Genomics
Genetic Analysis Facility

RESEARCH USE ONLY: The results contained in this report were generated in a lab that is not an accredited or licensed clinical laboratory. The results are intended for research purposes only. The underlying tests were not performed for the purposes of diagnosis, prophylaxis, or treatment.

Sample File	Sample Name	Panel	Marker	Allele 1	Allele 2
GP10-SYD22303	WT_Henle_Cells	GenePrint_10	AMEL	X	X
GP10-SYD22303	WT_Henle_Cells	GenePrint_10	CSF1PO	9	10
GP10-SYD22303	WT_Henle_Cells	GenePrint_10	D13S317	13.3	13.3
GP10-SYD22303	WT_Henle_Cells	GenePrint_10	D16S539	9	10
GP10-SYD22303	WT_Henle_Cells	GenePrint_10	D21S11	27	28
GP10-SYD22303	WT_Henle_Cells	GenePrint_10	D5S818	11	12
GP10-SYD22303	WT_Henle_Cells	GenePrint_10	D7S820	8	12
GP10-SYD22303	WT_Henle_Cells	GenePrint_10	TH01	7	7
GP10-SYD22303	WT_Henle_Cells	GenePrint_10	TPOX	8	12
GP10-SYD22303	WT_Henle_Cells	GenePrint_10	vWA	16	18

Uncropped Scans of Blots in Supplementary Figures

Supplementary Figure 2a

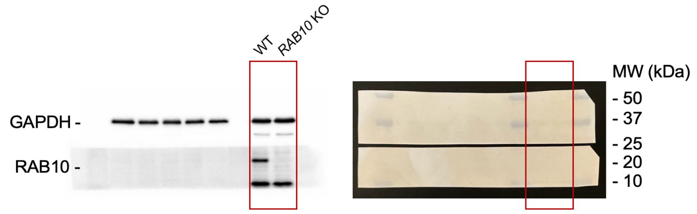

Supplementary Figure 2c

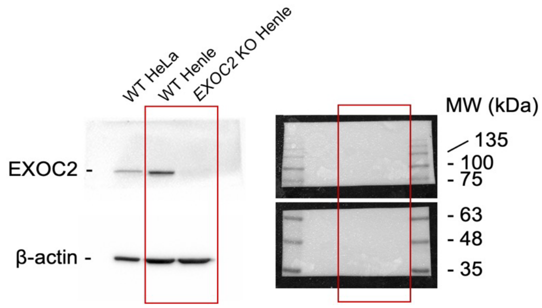

Supplementary Figure 2e

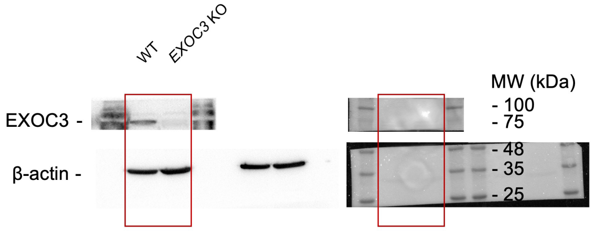

Supplementary Figure 2g

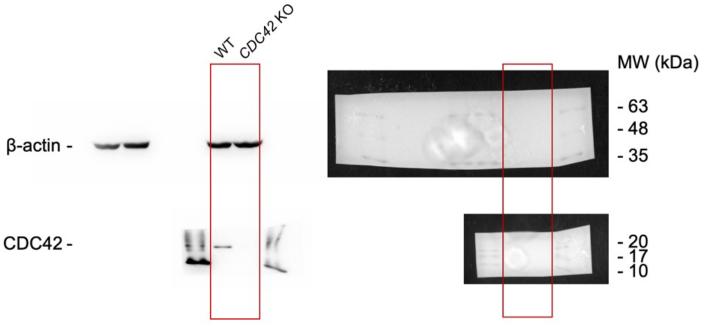

Supplement: Supplementary file 1 — Supplementary Information [file 41467_2024_47183_MOESM1_ESM.pdf]
