# Supplementary material for: Salmonella exploits membrane reservoirs for invasion of host cells
Source: Nat Commun. 2024 Apr 10;15:3120. doi: 10.1038/s41467-024-47183-x (PMC11006906; doi:10.1038/s41467-024-47183-x)
Supplement: Supplementary file 3 — Description of Additional Supplementary Files [file 41467_2024_47183_MOESM3_ESM.pdf]

Description of Additional Supplementary Files

Supplementary Data 1: RAB10 BioID interactome with high-confidence proximity interactors.
The 'BioID comparison' tab shows all the high confidence (BFDR<0.01) proximal interactors in at least one bait protein BioID experiment. The control BioID column displays the peptide counts of each given ID across the twelve replicates. For each RAB10 bait protein and each given ID, the peptide counts in the two biological and two technical replicates are shown, the peptide sum, and the SAINT values. Corresponding BFDR are shown in the 'Raw BioID' tab. For the mutant bait protein log2fc against WT, 1 was added to the peptide sums to avoid division by zero.

Supplementary Movie 1: RAB10^{+} tubule disassembly during *S*Tm infection.
Movie of WT Henle 407 cell transfected with GFP-RAB10 (green) and infected with WT BFP-*STm* (blue). RFP-LifeAct was used to identify invasion sites. Movie speed: 30 second per frame. Scale bar, 10 µm.

Supplementary Movie 2: Focused Ion Beam-Scanning Electron Microscopy volume of a WT Henle cell and 3D modeling of PM-derived RAB10^{+} membrane reservoir.
Movie of a FIB-SEM volume stack of a WT Henle 407 cell featured an intricate network of PM-derived RAB10^{+} membrane reservoirs. To assist with tracking of the reservoirs, the structures were 3D modeled (green) along with the plasma membrane (magenta).

Supplementary Movie 3: Correlated light and electron microscopy (CLEM) image of a WT Henle cell and RAB10^{+} membrane reservoir.
Movie of the full z-stack of a WT Henle 407 cell expressing GFP-RAB10 (green) and stained with CellMask (magenta) acquired using Airyscan microscopy. A portion of this cell was subsequently analyzed by FIB-SEM and the resulting volume was correlated to the light microscopy data.
