# Supplementary material for: Salmonella exploits membrane reservoirs for invasion of host cells
Source: Nat Commun. 2024 Apr 10;15:3120. doi: 10.1038/s41467-024-47183-x (PMC11006906; doi:10.1038/s41467-024-47183-x)
Supplement: Supplementary file 8 — Reporting Summary [file 41467_2024_47183_MOESM8_ESM.pdf]

nature portfolio

Corresponding author(s): John H. Brumell

Last updated by author(s): 2024/02/26

Reporting Summary

Nature Portfolio wishes to improve the reproducibility of the work that we publish. This form provides structure for consistency and transparency in reporting. For further information on Nature Portfolio policies, see our Editorial Policies and the Editorial Policy Checklist.

Statistics

For all statistical analyses, confirm that the following items are present in the figure legend, table legend, main text, or Methods section.

n/a	Confirmed	
☐	☒	The exact sample size (*n*) for each experimental group/condition, given as a discrete number and unit of measurement
☐	☒	A statement on whether measurements were taken from distinct samples or whether the same sample was measured repeatedly
☐	☒	The statistical test(s) used AND whether they are one- or two-sided<br>*Only common tests should be described solely by name; describe more complex techniques in the Methods section.*
☒	☐	A description of all covariates tested
☒	☐	A description of any assumptions or corrections, such as tests of normality and adjustment for multiple comparisons
☐	☒	A full description of the statistical parameters including central tendency (e.g. means) or other basic estimates (e.g. regression coefficient) AND variation (e.g. standard deviation) or associated estimates of uncertainty (e.g. confidence intervals)
☐	☒	For null hypothesis testing, the test statistic (e.g. *F*, *t*, *r*) with confidence intervals, effect sizes, degrees of freedom and *P* value noted<br>*Give P values as exact values whenever suitable.*
☒	☐	For Bayesian analysis, information on the choice of priors and Markov chain Monte Carlo settings
☒	☐	For hierarchical and complex designs, identification of the appropriate level for tests and full reporting of outcomes
☒	☐	Estimates of effect sizes (e.g. Cohen's *d*, Pearson's *r*), indicating how they were calculated

Our web collection on statistics for biologists contains articles on many of the points above.

Software and code

Policy information about availability of computer code

Data collection	For BioID-MS, HPLC was performed using a Proxeon EASY-nLC 1000 pump coupled with a Thermo Q-Exactive HF quadrupole-Orbitrap mass spectrometer.<br><br>For confocal imaging of IF samples (fixed), unless otherwise indicated, cells were imaged using a Quorum spinning disk microscope with a 10x or 20x, 1.0 NA objectives, or a 63x, 1.4 NA oil immersion objective (Leica DMI6000B inverted fluorescence microscope with a Yokogawa spinning disk head and Hamamatsu ORCA Flash 4 sCMOS camera) and Volocity 6.3 acquisition software (Quorum).<br><br>For confocal imaging of live cell samples, cells were imaged at 37°C using Leica DMI 6000B inverted fluorescence microscope with a Yokogawa spinning disk head and Hamamatsu ImagEM X2 camera.<br><br>For CLEM imaging, images were collected using a Zeiss LSM880 Airyscan microscope (light microscopy), and Zeiss Crossbeam 550 operating under SmartSEM (SEM) and Atlas 3D.<br><br>For AFM, measurements were performed using a Bioscope Resolve bioAFM system (Bruker) mounted on top of an Olympus IX-70 microscope base with Nanoscope software version 9.40.<br><br>For SEM, images were collected using a HITACHI FlexSEM 1000 II scanning electron microscope.<br><br>For WB, detection was performed using SuperSignal West Femto Maximum Sensitivity Substrate (Thermo).
Data analysis	BioID datasets were processed using Proteowizard, X!Tandem, and COMET against the human RefSeq Version 45 database. Proteins identified

Data analysis

with an iProphet cut-off of 0.9 and at least two unique peptides were analyzed with SAINT Express v.3.3.1. High confidence interactors were defined as those with a Bayesian false discovery rate (BFDR) ≤0.01.

For confocal images, the images were imported into Fiji v2.14.0 (ImageJ): For the percentage of cells containing at least one RAB10+ tubular membrane reservoir >10 µm in length (tubule length was measured by Fiji), cells were scored manually for the presence or absence of at least one Rab10+ tubule over 10 µm. For colocalization assays, the Mander's Correlation Coefficient (MCC) values were calculated for more than four images from each experiment by using Coloc 2, a Fiji plugin for colocalization analysis (http://imagej.net/Coloc_2). For RAB10's colocalization with PM markers on tubular membrane reservoirs, ROIs of single plane images were manually drawn to exclude the cell border. For other colocalization analysis, whole cell ROIs on maximum intensity z-projected images were chosen to represent the colocalization on the whole cell level. The line plot profiles were obtained by using the plot profile function in Fiji. For STm invasion site recruitment experiment, F-actin and STm staining (or Fluorescence-tagged STm) were used to denote the site of invasion at 10min p.i.. An enrichment of the indicated protein's signal at the STm invasion site, relative to the signal in the cytosol, was considered a positive recruitment. For invasion ruffle volume measurements, ROIs were drawn around individual invasion ruffles (containing one or more bacteria) volume measurements. For cell area measurements, the images were analyzed using a custom script in Python, available at https://github.com/DrSydor/RAB10KO_CellArea. Briefly, this script used a refined Cellpose cytosol model to segment the cells and measured the areas of those cells in which the average fluorescence intensity was above a pre-defined threshold. Details can be found in the manuscript's Methods section.

CLEM images were imported into Fiji v2.14.0 (ImageJ), where they were cropped and compiled into TIFF stacks. The stack alignment was refined using TrakEM257 and the images denoised using a structure preserving Gaussian denoising algorithm in Python. Fluorescence and EM images were correlated using the Icy 2.4.3.059 plug-in ec-CLEMv2. 3D modeling was performed using IMOD61 and final image and movie compilations were created in Adobe Illustrator and Premiere Pro, respectively.

AFM data (Young's modulus values) were determined by fitting the force curves using Indentation Analysis in NanoScope Analysis software version 1.90 with Contact Point Based fit and Hertzian (Spherical) model.

For SEM images, the images were then imported into Fiji v2.14.0 (ImageJ) and the individual invasion ruffle (containing one or more bacteria) were manually drawn to create ROIs for area measurements.

Processed images were imported and assembled in Adobe Illustrator v25.3.1 for labelling.
Statistical analyses were conducted using GraphPad Prism v9.0 and graphs were imported and assembled in Adobe Illustrator v25.3.1 for labelling.

For manuscripts utilizing custom algorithms or software that are central to the research but not yet described in published literature, software must be made available to editors and reviewers. We strongly encourage code deposition in a community repository (e.g. GitHub). See the Nature Portfolio guidelines for submitting code & software for further information.

Data

Policy information about availability of data

All manuscripts must include a data availability statement. This statement should provide the following information, where applicable:

- Accession codes, unique identifiers, or web links for publicly available datasets
- A description of any restrictions on data availability
- For clinical datasets or third party data, please ensure that the statement adheres to our policy

Raw mass spectrometry data for BioID analysis of RAB10 has been uploaded to the MassIVE public repository (https://massive.ucsd.edu/:) under accession #MSV000092081. Proximity interactions for FlagBirA"-RAB10 compared to 12x Flag-BirA* BioID alone runs performed under similar conditions ((1% FDR) are presented in Supplemental Table 1. Original Python code for the analysis of the cell areas is available at https://github.com/DrSydor/RAB10KO_CellArea. Source data are provided with this paper. Any additional information required to reanalyze the data reported in this paper is available from the lead contact upon request.

Research involving human participants, their data, or biological material

Policy information about studies with human participants or human data. See also policy information about sex, gender (identity/presentation), and sexual orientation and race, ethnicity and racism.

Reporting on sex and gender	Human research participants is not applicable to this study.
Reporting on race, ethnicity, or other socially relevant groupings	Human research participants is not applicable to this study.
Population characteristics	Human research participants is not applicable to this study.
Recruitment	Human research participants is not applicable to this study.
Ethics oversight	Human research participants is not applicable to this study.

Note that full information on the approval of the study protocol must also be provided in the manuscript.

Field-specific reporting

Please select the one below that is the best fit for your research. If you are not sure, read the appropriate sections before making your selection.

☒ Life sciences ☐ Behavioural & social sciences ☐ Ecological, evolutionary & environmental sciences

For a reference copy of the document with all sections, see nature.com/documents/nr-reporting-summary-flat.pdf

Life sciences study design

All studies must disclose on these points even when the disclosure is negative.

Sample size	No sample size calculation was performed and samples sizes were chosen based on common practice in the field and the variability within an experiment (Boddy et al., 2021).
Data exclusions	No data was excluded from the study.
Replication	Findings were reproduced at least 3 times with biological replicates.
Randomization	The experiments were not randomized. The experiments investigated specific cell types and treatments and required precise control over the experimental conditions, aspects that are not conditions amenable to randomization.
Blinding	The experiments were not blinded. The experiments investigated specific cell types and treatments and required precise control over the experimental conditions, aspects that are not conditions amenable to blinding.

Reporting for specific materials, systems and methods

We require information from authors about some types of materials, experimental systems and methods used in many studies. Here, indicate whether each material, system or method listed is relevant to your study. If you are not sure if a list item applies to your research, read the appropriate section before selecting a response.

Materials & experimental systems

n/a	Involved in the study	
☐	☒	Antibodies
☐	☒	Eukaryotic cell lines
☒	☐	Palaeontology and archaeology
☒	☐	Animals and other organisms
☒	☐	Clinical data
☒	☐	Dual use research of concern
☒	☐	Plants

Methods

n/a	Involved in the study	
☒	☐	ChIP-seq
☒	☐	Flow cytometry
☒	☐	MRI-based neuroimaging

Antibodies

Antibodies used

For immunofluorescence staining,
The following primary antibodies were used: mouse monoclonal anti-RAB10 (Sigma, SAB5300028, lot PM1009301) at a dilution of 1:100, mouse monoclonal anti-C-myc 9E10 (Thermo, MA1-980, lot XJ358688) at 1:500, mouse monoclonal anti-RAB4 (BD Biosciences, 610888, lot 606-259-1550) at a dilution of 1:100, rabbit polyclonal anti-RAB5 (Santa Cruz, sc-598, lot D0207) at a dilution of 1:100, rabbit polyclonal anti-RAB11 (Invitrogen, 71-5300, lot UD281527) at a dilution of 1:100, mouse monoclonal anti-EXOC7 (KeraFast, ED2001, lot 043019) at a dilution of 1:100, mouse monoclonal anti-GM130 (BD Biosciences, 610822, lot 8054546) at a dilution of 1:100, rabbit polyclonal anti-Salmonella (BD Transduction, 229481, lot 4017189) at a dilution of 1:100, chicken polyclonal anti-GFP (Rockland, 600-901-215S, lot 48932) at a dilution of 1:500, and rabbit polyclonal anti-RFP (Abcam, ab28664, lot 629768) at a dilution of 1:500.
The following secondary antibodies were used in this study: Alexa Fluor 488-conjugated goat anti-mouse IgG (Invitrogen, A-11029, lot 2179204), Alexa Fluor 488-conjugated goat anti-rabbit IgG (Invitrogen, A-11034, lot 2541675), Alexa Fluor 488-conjugated goat anti-chicken IgG (Invitrogen, A-32931, lot XB343360), Alexa Fluor 568-conjugated goat anti-mouse IgG (Invitrogen, A-11031, lot 2026148), Alexa Fluor 568-conjugated goat anti-rabbit IgG (Invitrogen, A-11011, lot 2379475), Alexa Fluor 647-conjugated goat anti-mouse IgG (Invitrogen, A-32728, lot XE344349), Alexa Fluor 647-conjugated goat anti-rabbit IgG (Invitrogen, A-32733, lot TL272452). The secondary antibodies were used at a dilution of 1:500.

For western blot,
The following primary antibodies were used: mouse monoclonal anti-RAB10 (Sigma, SAB5300028, lot PM1009301) at 1:1000, mouse monoclonal (6C5) anti-GAPDH (Millipore, MAB374, lot 3768063) at 1:10000, rabbit monoclonal anti-EXOC2 (Abcam, ab140620) at 1:1000, mouse monoclonal anti-beta tubulin (Sigma, T4026, lot 128M4790V) at 1:10000, rabbit polyclonal anti-EXOC3 (Proteintech, 14703-1-AP) at 1:1000 and rabbit polyclonal anti-CDC42 (Cell Signaling, 2462, lot 4) at 1:1000.
The following secondary antibodies were used: peroxidase-conjugated goat anti-rabbit IgG (Jackson ImmunoResearch, 11-035-144,

lots 152081 and 163676) or peroxidase-conjugated goat anti-mouse IgG (Jackson ImmunoResearch, 111-035-146, lot 157140)

Validation

All antibodies used in this study were validated by the manufacturers. Comprehensive validation statements, alongside relative details such as species reactivity, applications, and supporting citations, are accessible through the provided website links. Essential information pertaining to the antibodies employed in this study are outlined below:

mouse monoclonal anti-RAB10 (Sigma, SAB5300028)
Species reactivity: Mouse, Human
Application (in this study): IF (Immunofluorescence), WB (Western blot)
Other application: ELISA
Validation statements and other details (including citations): https://www.sigmaaldrich.com/CA/en/product/sigma/sab5300028

mouse monoclonal anti-C-myc 9E10 (Thermo, MA1-980)
Species reactivity: Human
Application (in this study): IF
Other application: WB, IHC, Flow, ELISA, IP, ChIP, FN, PLA, IV, Misc
Validation statements and other details (including citations): https://www.thermofisher.com/antibody/product/c-Myc-Antibody-clone-9E10-Monoclonal/MA1-980

mouse monoclonal anti-RAB4 (BD Biosciences, 610888)
Species reactivity: Rat, Human, Mouse, Dog
Application (in this study): IF
Other application: WB
Validation statements and other details (including citations): https://www.bdbiosciences.com/en-ca/products/reagents/microscopy-imaging-reagents/immunofluorescence-reagents/purified-mouse-anti-rab4.610888

rabbit polyclonal anti-RAB5 (Santa Cruz, sc-598)
Species reactivity: Human
Application (in this study): IF
Other application: WB
Validation statements and other details (including citations): https://www.scbt.com/p/rab-5b-antibody-a-20

rabbit polyclonal anti-RAB11 (Invitrogen, 71-5300)
Species reactivity: Dog, Human, Mouse, Non-human primate, Rabbit, Rat
Application (in this study): IF
Other application: WB, IHC, ELISA, IP, IM, Misc
Validation statements and other details (including citations): https://www.thermofisher.com/antibody/product/RAB11A-Antibody-Polyclonal/71-5300

mouse monoclonal anti-EXOC7 (KeraFast, ED2001)
Species reactivity: Human, Mouse, Rat
Application (in this study): IF
Other application: WB
Validation statements and other details (including citations): https://www.kerafast.com/item/579/anti-exocyst-complex-exo70-exoc7-subunit-70x13f3-antibody

mouse monoclonal anti-GM130 (BD Biosciences, 610822)
Species reactivity: Human, Mouse, Rat
Application (in this study): IF
Other application: WB, IHC, IP
Validation statements and other details (including citations): https://www.bdbiosciences.com/en-ca/products/reagents/microscopy-imaging-reagents/immunofluorescence-reagents/purified-mouse-anti-gm130.610822

rabbit polyclonal anti-Salmonella (BD Transduction, 229481)
Reactivity: Salmonella O Antiserum Group B Factors 1, 4, 5, 12
Application (in this study): IF
Other application: No
Validation statements and other details (including citations): https://www.fishersci.ca/shop/products/bd-diagnostic-systems-salmonella-shigella-typing-antisera-18/df2948476

chicken polyclonal anti-GFP (Rockland, 600-901-215S)
Reactivity: GFP, eGFP, rGFP
Application (in this study): IF
Other application: Dot Blot, ELISA, WB, IHC, Purification, Multiplex
Validation statements and other details (including citations): https://www.rockland.com/categories/primary-antibodies/gfp-antibody-600-901-215/

rabbit polyclonal anti-RFP (Abcam, ab28664)
Reactivity: RFP
Application (in this study): IF
Other application: WB
Validation statements and other details (including citations): https://www.abcam.com/products/primary-antibodies/rfp-antibody-ab28664.html
mouse monoclonal (6C5) anti-GAPDH (Millipore, MAB374
Species reactivity: Ca, H, M, R, Rb, F, Fe, Po

Application (in this study): WB
Other application: ELISA, IP, ICC, IF, IHC
Validation statements and other details (including citations):
https://www.emdmillipore.com/CA/en/product/Anti-Glyceraldehyde-3-Phosphate-Dehydrogenase-Antibody-clone-6C5,MM_NF-MAB374

rabbit monoclonal anti-EXOC2 (Abcam, ab140620)
Species reactivity: Human
Application (in this study): WB
Other application: Flow Cyt (Intra)
Validation statements and other details (including citations): https://www.abcam.com/products/primary-antibodies/exoc2-antibody-epr9420-ab140620.html

rabbit polyclonal anti-EXOC3 (Proteintech, 14703-1-AP)
Species reactivity: Human, Mouse, Rat
Application (in this study): WB
Other application: IHC, IF, ELISA
Validation statements and other details (including citations): https://www.ptglab.com/products/EXOC3-Antibody-14703-1-AP.htm

mouse monoclonal anti-beta tubulin (Sigma, T4026)
Species reactivity: Human, Rat, Frog, Moth, Mouse, Plant, Rabbit, Chicken, Bovine, Wheat, Sea urchin, Hamster
Application (in this study): WB
Other application: FACS, IF
Validation statements and other details (including citations):
https://www.sigmaaldrich.com/CA/en/product/sigma/t4026

rabbit polyclonal anti-CDC42 (Cell Signaling, 2462)
Species reactivity: Human, Mouse, Rat, Monkey, Bovine
Application (in this study): WB
Other application: No
Validation statements and other details (including citations):
https://www.cellsignal.com/products/primary-antibodies/cdc42-antibody/2462

Eukaryotic cell lines

Policy information about cell lines and Sex and Gender in Research

Cell line source(s)	Henle 407 cells (ATCC, CCL-6), MCF-7 cells (ATCC, HTB-22), Caco-2 cells (ATCC, HTB-37), T84 cells (ATCC, CCL-248), HeLa cells(ATCC, CCL-2), SH-SY5Y cells (ATCC, CRL-2266), HEK293 cells (ATCC, CRL-1573) and MEF cells (ATCC, SCRC-1008) wereobtained from the American Type Culture Collection (ATCC).<br>RAB10 KO Henle 407 cells were used and previously described (Boddy et al., 2021).<br>CDC42 KO Henle 407 cells were used and previously described (Walpole et al., 2022).<br>EXOC2 and EXOC3 KO Henle 407 cells were generated and described in this study.
Authentication	The cells lines from ATCC were authenticated by ATCC. The Henle cell line was further authenticated by the SickKids BioBank via STR profiling using the GenePrint 10 System.
Mycoplasma contamination	All cell lines used were tested negative for mycoplasma contamination.
Commonly misidentified lines<br>(See ICLAC register)	No commonly misidentified cell lines were used in the study.

Plants

Seed stocks	N/A- This study did not involve seed stocks.
Novel plant genotypes	N/A- This study did not involve plants of any sort, novel or otherwise.
Authentication	N/A- For the reasons outlines above.
